# The *ADORA2A* TT Genotype Is Associated with Anti-Inflammatory Effects of Caffeine in Response to Resistance Exercise and Habitual Coffee Intake

**DOI:** 10.3390/nu15071634

**Published:** 2023-03-28

**Authors:** Mohammad Rahman Rahimi, Ekaterina A. Semenova, Andrey K. Larin, Nikolay A. Kulemin, Edward V. Generozov, Beata Łubkowska, Ildus I. Ahmetov, Hadi Golpasandi

**Affiliations:** 1 Department of Exercise Physiology, University of Kurdistan, Sanandaj 66177-15175, Iran; 2 Department of Molecular Biology and Genetics, Federal Research and Clinical Center of Physical-Chemical Medicine of Federal Medical Biological Agency, 119435 Moscow, Russia; 3 Research Institute of Physical Culture and Sport, Volga Region State University of Physical Culture, Sport and Tourism, 420138 Kazan, Russia; 4 Faculty of Physical Culture, Gdansk University of Physical Education and Sport, 80-336 Gdansk, Poland; 5 Laboratory of Genetics of Aging and Longevity, Kazan State Medical University, 420012 Kazan, Russia; 6 Department of Physical Education, Plekhanov Russian University of Economics, 115093 Moscow, Russia; 7 Research Institute for Sport and Exercise Sciences, Liverpool John Moores University, Liverpool L3 5AF, UK

**Keywords:** adenosine, inflammation, immune system, neutrophils, strength, sports, performance, training

## Abstract

Caffeine is an adenosine A_2A_ receptor (*ADORA2A*) antagonist with ergogenic and anti-inflammatory effects. Previous studies have reported that the *ADORA2A* gene regulates glutamate metabolism and immune responses, with the *ADORA2A* rs5751876 TT genotype (with high sensitivity to caffeine) showing larger ergogenic effect following caffeine ingestion. We therefore hypothesized that the TT genotype would be associated with greater anti-inflammatory effects of caffeine in response to exercise, and with higher coffee intake in physically active individuals. The aim of the present study was twofold: (1) to investigate the association of the *ADORA2A* variant with the anti-inflammatory effects of caffeine in response to intense resistance exercise (RE), and (2) to analyze the association of the rs5751876 with coffee intake in physically active individuals (*n* = 134). Fifteen resistance-trained athletes participated in a randomized, double-blind, placebo-controlled cross-over study, where they consumed 6 mg/kg of caffeine or placebo one hour prior to performing an RE protocol. Blood samples were taken immediately from the arterial vein before, immediately after, and 15 min after RE for the analysis of inflammatory markers myeloperoxidase (MPO) and acetylcholinesterase (AChE). We found that the *ADORA2A* TT genotype carriers experienced lower exercise-induced inflammatory responses (*p* < 0.05 for AchE) when compared to the C allele carriers (i.e., CC/CT) one hour following the ingestion of caffeine. Furthermore, the *ADORA2A* TT genotype was positively associated with coffee intake (*p* = 0.0143; irrespective of *CYP1A2* rs762551 polymorphism). In conclusion, we found that the *ADORA2A* gene polymorphism is associated with anti-inflammatory effects of caffeine in response to resistance exercise, as well as with habitual coffee intake in physically active individuals.

## 1. Introduction

Training, nutrition, motivation, and genetics (talent) are key factors in achieving high levels of athletic performance [1]. Recently, attention has been paid to the role of genetic factors, and in particular performance-enhancing DNA polymorphisms, among sports scientists and physiologists. More than 220 polymorphisms have been reported to be associated with athlete status [2]. Furthermore, previous studies have indicated that genetic polymorphisms are associated with individual responses to nutrients [3,4,5], oxidative stress [6,7,8,9,10], inflammatory factors [11], and cardiovascular parameters during exercise [12].

Adenosine is one of the metabolites produced by dephosphorylation of the Adenosine Triphosphate (ATP) via 5-nucleotidease enzyme during the exercise [13]. Adenosine influences through the adenosine receptors such as A_1_, A_2A_, A_2B_, and A_3_ [14,15]. In general, adenosine as an inhibitory molecule plays an important role in nerve stimulation and synaptic transmission of the brain [16]. The A_2A_ receptor (A2AR) may influence the motor function because A2ARs are widely expressed on striatum neurons affecting the basal ganglia and contributing to motion control and motivation [17].

Research suggests that caffeine can block adenosine receptors, which may induce increased neurotransmitter dopamine release [18] and improve the transfer of dopamine D2 to the striatum, with motor activity change [19]. Davis et al. [20] showed that caffeine can delay fatigue through central nervous system mechanisms, at least in part by blocking adenosine receptors. Also, less appetite to caffeine was reported in rats lacking adenosine A2AR when compared to those receiving this receptor [21].

Adenosine A_1_ and A_2A_ receptors are expressed in many tissues, including brain, skeletal muscle, kidney, spinal cord, adipose tissue, and immune cells such as neutrophils. Blocking A_1_ receptors by adenosine at picomolar concentrations increases the chemotaxis and binding the polymorphonuclear cells [22]; as such, it causes a pro-inflammatory effect. In contrast, at micromolar concentrations, adenosine has been shown to inhibit the production of superoxide anions through the occupation of adenosine A2ARs [23]. Caffeine, on the other hand, is an adenosine analogue increasing the cytosolic concentration of cAMP by influencing on A_1_ and A_2A_ receptors [24].

There seems to be inter-individual differences in response to caffeine intake among people [3,25,26,27]. These inter-individual differences regarding the caffeine intake and response to it have been observed in individuals with genetic variants in the adenosine A_2A_ receptor gene (*ADORA2A*; regulates glutamate metabolism and immune responses) rs5751876 polymorphism [25]. In this polymorphism, Cytosine (C) is replaced by Thymidine (T) in nucleotide number 1976 (1976C > T; with T allele frequencies of 68.2, 45.8, 52.8, 39.1, and 66.4% in Africans, Americans, East Asians, Europeans, and South Asians, respectively) [25]. Interestingly, Loy et al. [28] found improvement in bicycling performance following the caffeine supplementation in subjects with *ADORA2A* TT (high sensitivity to caffeine) genotype, but no improvement was observed in C allele carriers (low or moderate sensitivity to caffeine) [28]. It should be noted that other polymorphisms located in the *ADORA2A* and Adenosine A1 Receptor (*ADORA1*) genes have been also reported [29]. Furthermore, previous studies have reported that the AA genotype (predicts fast metabolism of caffeine) of the Cytochrome P450 Family 1 Subfamily A Member 2 (*CYP1A2*; inhibited by caffeine and induced by tobacco) gene rs762551 polymorphism is associated with higher coffee intake and larger ergogenic effect following caffeine ingestion [3,4]. Therefore, when analyzing the association of the *ADORA2A* genotype with caffeine-related phenotypes, it is necessary to consider the *CYP1A2* rs762551 genotype.

There is no study investigating the effect of the *ADORA2A* polymorphism on inflammatory markers following exercise, but previous studies have reported the increase in inflammatory factors following exercise [30,31]. Strenuous physical activity can result in degranulation of neutrophils, which increases plasma concentrations of neutrophilic enzymes such as Myeloperoxidase (MPO) and elastase [30,31,32]. The MPO enzyme is stored in the atherophylic granules of the naive neutrophil cells [33], playing a vital role in inflammatory processes and oxidative stress. Neutrophils and activated monocytes are the main sources of plasma MPO production.

In addition to MPO, the hydrolytic acetylcholinesterase (AchE) enzyme is considered as an inflammatory marker [34,35]. Increased activity of AchE enzyme has been reported after intense exercise in rats [35]; this enzyme binds to the membrane of erythrocytes, platelets, leukocytes, and endothelial cells, leading to the breakdown of acetylcholine. Previous studies have shown that acetylcholine has an anti-inflammatory effect and suppresses the production of inflammatory cytokines [36]. Thus, the level of acetylcholine reduces as a result of increased activity of the AchE enzyme, which reduces the anti-inflammatory function of acetylcholine [34].

It has been shown that caffeine has anti-inflammatory effects and is able to significantly reduce the activity of MPO and AchE during exercise activity [35,37]. However, the importance of the adenosine A2AR and the anti-inflammatory role of caffeine have not been investigated in any research; specifically, there is no study investigating the resistance exercise-induced inflammatory responses (MPO and AchE) to caffeine ingestion in athletes with different genotypes of the *ADORA2A* gene polymorphism. There is also no study reporting the association between the *ADORA2A* genotypes and habitual coffee intake in physically active individuals.

Therefore, the aim of the present study was twofold: (1) to investigate the association of the *ADORA2A* variant with the anti-inflammatory effects of caffeine in response to intense resistance exercise (RE), and (2) to analyze the association of the rs5751876 with coffee intake in physically active individuals.

## 2. Materials and Methods

### 2.1. Ethics Statement

The Institutional Review Board of the Faculty of Humanities and Social Science (No. 1314796, 08.10.95) and Iran National Committee for Ethics in Biomedical Research at University of Kurdistan (IR.UOK.REC.1398.047) and the Ethics Committee of the Federal Research and Clinical Center of Physical-Chemical Medicine of the Federal Medical and Biological Agency of Russia (Approval number 2017/04) approved the protocols for the research. Written informed consent was obtained from each participant. The studies were complied with the guidelines set out in the Declaration of Helsinki and ethical standards in sport and exercise science research.

### 2.2. Participants

#### 2.2.1. Resistance Exercise Study

Fifteen resistance-trained Iranian men were randomly selected to participate in the randomized, double-blind, placebo-controlled study. Physical characteristics of 15 male subjects with different *ADORA2A* genotypes are shown in Table 1. At first, athletes were invited to participate in the briefing session with the presence of the researcher. After complete explanation of the research objectives and methods of measurement, participants signed a written informed consent form and completed a health questionnaire and a caffeine consumption questionnaire. Eligible participants were athletes who had not used any anti-inflammatory drug or performance-enhancing supplements during the previous 3 months, and they were light caffeine consumers (more than 50 mg/day), as previously proposed [38,39].

#### 2.2.2. Habitual Coffee Intake Study

A total of 134 Russian healthy (non-smokers), physically active (>3 training sessions per week) subjects (45 females, age 28.4 (7.1) years, height 168.0 (5.9) cm, weight 59.5 (6.1) kg; 89 males, age 31.4 (8.6) years, height 179.8 (6.2) cm, weight 80.2 (10.3) kg) participated in this study.

### 2.3. Resistance Exercise Study Design

One week before starting the experimental session, maximal strength was assessed by one Repetition Maximum (1RM) test in bench press, leg press, seated cable row, and shoulder press. Participants were asked to abstain from performing the exercise 3 days before the 1RM test and experimental sessions, and to avoid consumption of caffeine for 24 h before testing.

Then, in a randomized, double-blind, placebo-controlled, and cross-over design, participants consumed 6 mg per kg bodyweight caffeine or a placebo one hour before RE. After one hour, blood samples were taken from antecubital vein of the subjects; then, they participated in 3 sets of RE to failure with 85% of 1RM, consisting of bench press, leg press, seated cable row, and shoulder press. Two minutes of rest period between sets and exercise was taken. Additional blood samples were taken from the venous vein immediately after (Post) and 15 min after RE (15 min Post). After 3 days of washout, the subjects were recalled and treated with caffeine or placebo, with a change in supplementation and placebo, under the same conditions. Then, they participated in RE under the previous conditions again.

### 2.4. Assessment of Habitual Coffee Intake

Habitual coffee intake was assessed using a dietary questionnaire administered to all participants. Responses to the questions on coffee were assigned values for frequency per week (never = 0, once per week = 1, 2–3 times per week = 3, 4–5 times per week = 5, and once or more a day = 7).

### 2.5. Biochemical Analysis

After sampling, 5 mL of venous blood was used for serum separation. Serum samples were stored at −20 °C until the day of analysis. The ELISA method was used for analyzing the activity of myeloperoxidase (MPO) and acetylcholinesterase enzymes (AchE) in Iranian subjects (ELISA Kit, Eastbiopharm, Hangzhou, Zhejiang, China).

### 2.6. Genotyping

#### 2.6.1. Resistance Exercise Study

After sampling, 2 mL of blood was injected in K2EDTA-containing Venoject for DNA extraction (TIANamp Genomic DNA) to study the Single-Nucleotide Polymorphism (SNP) in *ADORA2A* rs5751876 polymorphism. The genotyping in *ADORA2A* polymorphism was analyzed through the Amplification of Refractory Mutation System–Polymerase Chain Reaction (ARMS–PCR) [40]. In this method, the reaction is carried out in two separate tubes, one of which contains mutated primers and the other contains wild primers. If proliferation is carried out in a tube containing a mutated primer, a mutation has occurred in the target DNA, and proliferation has occurred in a tube containing a typical primer, indicating that no mutation has occurred. The primers used include 5-TGAGCGGAGGCCCAATGGCAAC-3 to detect C allele and 5-TGAGCGGAGGCCCAATGGCAAT-3 to detect T allele and return primer of 5-CTGGCACTGCTCTGTTACAACTCC-3, which were added per 25 μL of PCR mixture. Primers were placed in a Thermal Cycler to perform the PCR reaction.

The thermal protocol for PCR operation consisted of step (1) the primary denaturation for 10 min at 94 °C, step (2) second denaturation for 1 min at 94 °C, step (3) annealing for 1 min at 65 °C, step (4) extension for 1 min at 72 °C (steps 2 to 4, 32 cycles), and step (5) final extension for 5 min at 72 °C.

In order to verify that PCR was performed in all samples, the internal control was used, in which two primers of mitochondrial genome (namely L strand: 5′-CTCCACCATTAGCACCCAAAGC-3′ and H strand: 5′-CCTATTTGTTTATGGGGTGATG-3′) were used to produce a 250 bp fragment. All samples were run in duplicate with two negative controls.

#### 2.6.2. Habitual Coffee Intake Study

Molecular genetic analysis was performed with DNA samples obtained from leukocytes (venous blood). Four ml of venous blood were collected in tubes containing EDTA (Vacuette EDTA tubes, Greiner Bio-One, Kremsmünster, Austria). DNA extraction and purification were performed using a commercial kit according to the manufacturer’s instructions (Technoclon, Moscow, Russia). HumanOmniExpressBeadChips (Illumina Inc., San Diego, CA, USA) or HumanOmni1-Quad BeadChips (Illumina, San Diego, CA, USA) were used to genotype *ADORA2A* rs5751876 and *CYP1A2* rs762551 polymorphisms, respectively, as previously described [41,42].

### 2.7. Statistical Analysis

Statistical analyses were conducted using Statistical Package for the Social Sciences (SPSS) for Windows v21.0 (IBM Crop, Armonk, NY, USA) or GraphPad InStat (GraphPad Software, Inc., San Diego, CA, USA) software. A 2 × 2 × 3 general linear model repeated measures ANOVA was used to examine acetylcholinesterase and myeloperoxidase concentrations after caffeine ingestion, with genotype group (TT vs. CT/CC) as between-subjects factor and condition (caffeine vs. placebo) and sampling time (pre, post, and 15 min post) as the within-subjects factor. Multiple regression was used to assess the relationship between coffee intake and *ADORA2A* polymorphism (adjusted for sex, age, and *CYP1A2* rs762551 genotype (predictor of caffeine intake)). All data are presented as mean (SD). *p* values < 0.05 were considered statistically significant.

## 3. Results

### 3.1. Association of ADORA2A Variant with Anti-Inflammatory Effects of Caffeine in Response to Intense Resistance Exercise

**Figure 1:**
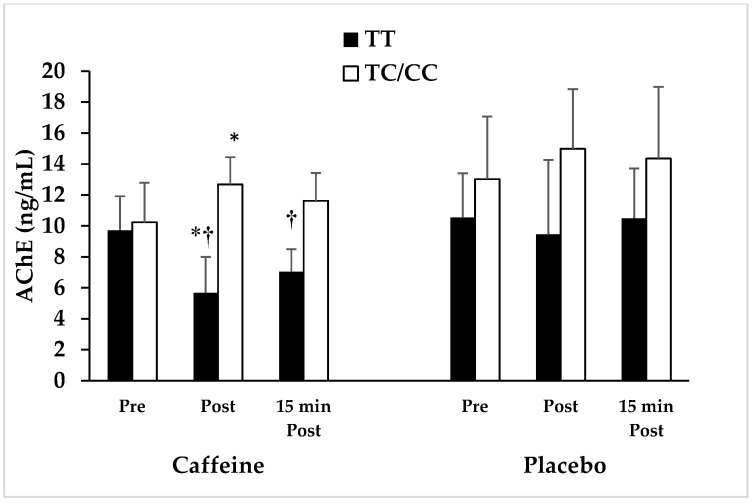
AChE response to resistance exercise in athletes with *ADORA2A* C allele and TT genotype following caffeine and placebo supplementation. * indicates significant difference vs. pre (*p* = 0.043). ^†^ indicates significant differences between TT genotype and C allele carriers (*p* = 0.013). AChE: acetylcholinesterase.

**Figure 2:**
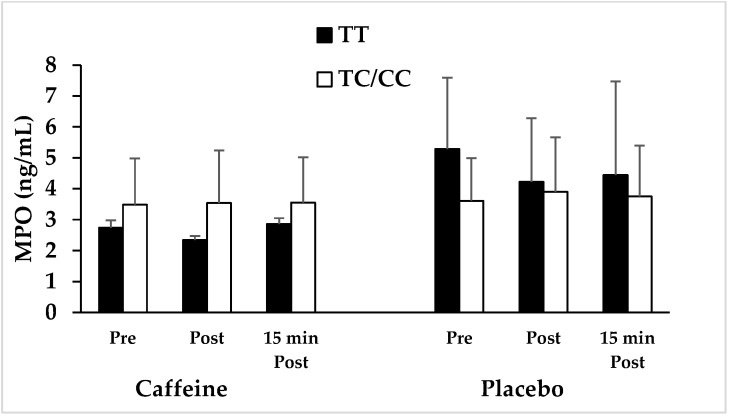
MPO response to resistance exercise in athletes with *ADORA2A* C allele and TT genotype following caffeine and placebo supplementation. No significant differences in the changes have been observed. MPO: myeloperoxidase.

No significant differences were determined in age, height, weight, soft lean mass, body fat mass, percent body fat, body mass index, and basal metabolic rate between the carriers of the *ADORA2A* TT genotype and C allele (i.e., CT/CC genotypes) (Table 1). The role of *ADORA2A* genotype on AChE and MPO is shown in Figure 1 and Figure 2 and Table 2. A 2 × 2 × 3 general linear model repeated measures ANOVA was used to examine AChE enzyme concentrations after caffeine ingestion, with genotype group (TT vs. CT/CC) as between-subjects factor and condition (caffeine vs. placebo) and sampling time (pre, post, and 15 min post) as the within-subjects factor.

Repeated measures ANOVA revealed a significant genotype (TT vs. CT/CC) × time (pre, post, and 15 min post) interaction effect in caffeine condition (F = 8.36, *p* = 0.013, ƞ^2^ = 0.39), in which the C allele carriers of the *ADORA2A* gene had significantly higher AChE enzyme concentrations than the TT genotype in caffeine condition at Post (39.95%) and 15 min Post RE (32.95%). However, there were no significant genotype (TT vs. CT/CC) × time (pre, post and 15 min post) interaction effects in placebo condition (F = 2.20, *p* = 0.161, ƞ^2^ = 0.145), in which the C allele carriers tended to have higher AChE enzyme concentrations than the TT genotype in placebo condition at Post (15.35%) and 15 min Post RE (19.08%). The comparison of AChE enzyme concentrations within-subjects demonstrated the significant effect of time in caffeine condition (F = 4.27, *p* = 0.043, ƞ^2^ = 0.24) and a non-significant effect of time in placebo condition (F = 0.031, *p* = 0.96, ƞ^2^ = 0.002).

Regarding to MPO concentration, no significant genotype (TT vs. CT/CC) × time (pre, post, and 15 min post) interaction effect in both caffeine (F = 1.55, *p* = 0.23, ƞ^2^ = 0.107) and placebo (F = 0.81, *p* = 0.38, ƞ^2^ = 0.059) condition was observed (Figure 2 and Table 2). However, MPO concentration tended to be higher in C allele carriers than the TT genotype in caffeine condition at Post (33.89%) and 15 min Post RE (19.43%).

### 3.2. Association of ADORA2A Variant with Habitual Coffee Intake

In 134 physically active Russian individuals (TT: 22, CT: 53, CC: 59), the *ADORA2A* gene rs5751876 polymorphism met Hardy–Weinberg expectations (*p* > 0.05). The *ADORA2A* TT genotype was positively associated with coffee intake in physically active subjects (females: TT 4.4 (3.6) times per week, CT + CC 3.4 (3.0) times per week; males: TT 3.9 (2.9) times per week, CT + CC 2.7 (2.7) times per week; *p* = 0.0143 adjusted for age, sex, and *CYP1A2* rs762551 genotype).

## 4. Discussion

In recent decades, the role of different genetic polymorphisms in inter-individual differences in nutrients and exercise responses has been taken into consideration by exercise and sports scientists [3,4,5,27,28,43,44,45]. Regarding the inter-individual differences in response to caffeine, previous studies have examined the effects of common polymorphisms in the *CYP1A2* and *ADORA2A* genes; the results pertinent to the role of *ADORA2A* polymorphism on the ergogenic effects of caffeine are limited to one study [28]. The authors have observed the improvement of exercise performance in female subjects with TT (high sensitivity to caffeine) genotype consuming 5 mg/kg of caffeine when compared to a placebo; however, no improvement was observed in carriers of the C allele [28]. Despite this, there is no research on the effect of *ADORA2A* gene polymorphism on the response of the degranulation of neutrophils and the AChE hydrolytic enzyme as a result of caffeine supplementation in athletes. Previous research investigated the effect of the polymorphism of 8-oxoguanine DNA glycosylase-1 (*OGG1*) and Glutathione S-transferase M1 (*GSTM1*) genes on the oxidative stress response to intense exercise; the results showed that both polymorphisms alter oxidative stress responses to exercise [7,8]. Therefore, it is also likely that *ADORA2A* polymorphism may modulate the anti-inflammatory effects of caffeine during strenuous exercise.

MPO is one of the most common markers of neutrophil degranulation. The concentration of MPO in the blood circulation depends on the intensity of exercise; its concentration is higher in cases of exercise performed with an intensity of 85% VO_2max_ when compared to 65% VO_2max_ [46]. Furthermore, a significant increase was observed in MPO concentration after intense exercise [31], swimming in rats, and running on treadmills in humans [30] when compared to baseline value. Therefore, applying strategies for reducing neutrophil degranulation during intense exercise can help boost the immune system against pathogens because the neutrophil degranulation leads to a decrease in the neutrophil capacity required for phagocytosis by these cells.

In the present study, there was no significant difference in MPO concentration in caffeine consumption versus placebo between TT genotype and C allele carriers. However, in the case of caffeine consumption, there were fewer changes in the percentage of MPO concentration in subjects with TT genotype when compared to the placebo at all three stages. In addition, similar changes were observed in carriers of C allele in terms of MPO concentration at all three measurement steps; however, these changes were less pronounced than in TT genotype carriers. It should be noted that the observed reduction in the concentration of MPO could be indicative of the anti-inflammatory effects of caffeine. the statistically insignificant results are possibly due to the low number of participants in the research. Previous studies have also shown that consumption of 6 mg/kg of caffeine resulted in decreased plasma MPO activity in rats. In case of rats, it was also revealed that taking 6 mg of caffeine along with 4 weeks of swimming training led to a decrease in plasma MPO activity after exercise [37]. Stefanello et al. [35] also demonstrated that caffeine intake reduced the activity of plasma MPO after exercise [35].

The hydrolytic AChE enzyme, which is attached to the membrane of erythrocytes, platelets, leukocytes, and endothelial cells, has an inflammatory effect [34]. The results of our study showed that there is a significant difference in AChE concentration in caffeine consumption conditions between the carriers of TT and CT/CC genotypes. In other words, caffeine intake one hour before the RE significantly decreased the concentration of AChE immediately after and 15 min after the resistance exercise in athletes with TT genotype when compared to those with the C alleles. Within the subjects, changes indicated a significant decrease in the concentration of the AChE hydrolytic enzyme in the TT genotype and a significant increase in AchE immediately after RE when compared to the pre-RE in placebo condition. Our findings are consistent with the results of previous studies, whic showed a significant reduction in AChE activity in Wistar rats as a result of consuming 6 mg/kg of caffeine [37]. In another study, the results showed that consuming 6 mg/kg of caffeine reduced AChE activity in trained rats [34].

Caffeine consumption in athletes with TT genotype resulted in 5%, 55%, and 39% decreases in AChE enzyme concentrations before, immediately after, and 15 min after RE compared to placebo, respectively. Nevertheless, caffeine intake in athletes carrying C allele resulted in less significant reductions in AChE concentration, which were equal to 18% (*p* = 0.19), 36% (*p* = 0.28), and 26% (*p* = 0.25) before, immediately after, and 15 min after RE compared to placebo, respectively. Seemingly, the anti-inflammatory effects of caffeine are higher in athletes with TT genotype in *ADORA2A* gene compared to C allele carriers. As previously stated, the AChE hydrolytic enzyme has an inflammatory effect; thus, reducing the concentration of this enzyme can lead to an increase in acetylcholine levels, which has anti-inflammatory effects and suppresses the production of inflammatory cytokines [36].

Caffeine exerts anti-inflammatory effects through antagonizing adenosine receptors, especially the A2AR, which are involved in inflammatory processes [47]. In addition, caffeine may suppress the expression of cytokines in immune cells by blocking the Inositol-3-Phosphate (I3PR) Receptors [48].

Since the AChE enzyme is linked to the leukocyte membrane, it is suggested that the expression and activity of this enzyme be investigated following the use of caffeine at the level of immune cells (leukocytes) in individuals with TT, CT, and CC genotypes of the *ADORA2A* gene. In addition, the AChE hydrolytic enzyme is considered to be one of the key enzymes in the neuromuscular junction (NMJ) and terminating neurotransmission by acetylcholine hydrolysis; it is also one of the key enzymes contributing to the fatigue caused by exercise. Therefore, we suggest investigating the effect of caffeine on the expression and activity of AChE in NMJ in response to intense exercise in animal studies. We also suggest conducting future studies on the effect of *ADORA2A* polymorphism on the anti-inflammatory and ergogenic properties of caffeine with a larger sample size across the two genders and various sports.

We have also identified that *ADORA2A* TT genotype carriers consumed coffee significantly more frequently than C allele carriers among healthy and physically active individuals with non-smoking status (adjusted for age, sex, and *CYP1A2* genotype). This is in line with UK Biobank data, where T allele has been reported to be positively associated with coffee intake in 334,659 subjects (*p* = 0.00036) [49]. It should be noted, however, that in the previous study, involving 2735 participants with the risk of myocardial infarction (and some were smokers), the TT genotype was associated with less caffeine consumption [26]. One might suggest that the discrepancies in the results are due to the differences in the cohort (health and physical activity statuses) and using *CYP1A2* rs762551 genotype as a covariate in our study (rs762551 A allele is a strong predictor of higher caffeine intake according to the UK Biobank data [49]).

There are some limitations that should be acknowledged. First, the size of two independent cohorts was from small (resistance exercise study) to moderate (coffee intake study). Thus, although our results were further partly supported (UK Biobank data, *n* = 334,659), extension of and replication within groups of differing geographic ancestry is needed to translate these findings more broadly. Second, in addition to the measured AChE and MPO biomarkers, our findings would receive more support from the analysis of anti-inflammatory cytokines such as IL-4, IL-6, IL-9, IL-10, IL-11, IL-13, and IL-19. Third, functional studies are necessary to establish a causal relationship between the *ADORA2A* genotype and anti-inflammatory effects of caffeine in response to resistance exercise.

In conclusion, we found that the *ADORA2A* gene rs5751876 polymorphism is associated with anti-inflammatory effects of caffeine in response to resistance exercise, as well as with habitual coffee intake in physically active individuals.

## Figures and Tables

**Table 1:** Physical characteristics of the subjects with *ADORA2A* C allele (*n* = 10) and TT genotype (*n* = 5).

Genotype Group	TT (*n* = 5)		CT/CC (*n* = 10)		T	*p* Value
	M	SD	M	SD		
Age (years)	21.75	3.30	20.20	1.87	1.13	0.28
Height (cm)	179.5	3.69	178.5	4.67	0.38	0.71
Weight (kg)	73.87	6.54	72.47	10.38	0.24	0.80
Soft Lean Mass (kg)	60.55	4.25	60.38	7.26	0.04	0.96
Body Fat Mass (kg)	9.97	2.90	8.73	4.34	0.52	0.61
% Body Fat	13.32	3.15	11.71	4.50	0.64	0.52
Body mass index (kg/m^2^)	22.90	1.08	22.80	3.59	0.05	0.95
Basal Metabolic Rate	2026.70	183.64	2055.40	193.56	−0.25	0.80

Values are Mean ± SD, there were no significant differences between group.

**Table 2:** Percent change of MPO and AChE levels in caffeine and placebo condition in *ADORA2A* C allele and TT genotype carriers.

Trait	Group	TT Genotype (*n* = 5)						CT/CC Genotype (*n* = 10)					
		Pre	∆	Post	∆	15 m Post	∆	Pre	∆	Post	∆	15 m Post	∆
MPO (ng/mL)	C	2.7 ± 0.2	48.1 ↓	2.3 ± 0.1	44.5 **↓**	2.9 ± 0.2	35.6 ↓	3.5 ± 1.5	3.6 **↓**	3.5 ± 1.7	9.2 **↓**	3.6 ± 1.5	5.3 ↓
	P	5.3 ± 2.3		4.2 ± 2.1		4.4 ± 3.0		3.6 ± 1.4		3.9 ± 1.8		3.8 ± 1.6	
AChE (ng/mL)	C	9.7 ± 2.2	5.1 ↓	5.7 ± 2.3	55.2 ↓ *	7.0 ± 1.5	39.4 ↓ *	10.6 ± 2.6	19.0 ↓	9.5 ± 1.8	36.8 ↓ *	10.5 ± 1.8	26.9 ↓
	P	10.2 ± 2.9		12.7 ± 4.8		11.6 ± 3.2		13.0 ± 4.1		15.0 ± 3.2		14.4 ± 4.6	

C, caffeine group; P, placebo group. * *p* < 0.05, statistically significant changes. ∆, change in %; ↓, decrease; MPO, myeloperoxidase; AChE, acetylcholinesterase.

## Data Availability

The data presented in this study are available on request from the corresponding authors.
